# Supplementary material for: Experimental–Density Functional Theory (DFT) Study of the Inhibitory Effect of Furan Residues in the Ziegler–Natta Catalyst during Polypropylene Synthesis
Source: Int J Mol Sci. 2023 Sep 21;24(18):14368. doi: 10.3390/ijms241814368 (PMC10532384; doi:10.3390/ijms241814368)
Supplement: Supplementary file 1 [file ijms-24-14368-s001.zip › ijms-2592566-supplementary.pdf]

Supporting Information for

Experimental-density functional theory (DFT) study of the inhibitory effect of furan residues in the Ziegler-Natta catalyst during polypropylene synthesis

Joaquín Hernández-Fernández [1,2,3,*], Esneyder Puello-Polo [4] and Edgar Marquez [5,*]

[1] Chemistry Program, Department of Natural and Exact Sciences, San Pablo Campus, University of Cartagena, Cartagena 130015, Colombia

[2] Chemical Engineering Program, School of Engineering, Universidad Tecnológica de Bolivar, Parque In-dustrial y Tecnológico Carlos Vélez Pombo Km 1 Vía Turbaco, Cartagena 130001, Colombia

[3] Department of Natural and Exact Science, Universidad de la Costa, Barranquilla 080002, Colombia

[4] Group de Investigación en Oxi/Hidrotratamiento Catalítico Y Nuevos Materiales, Programa de Química-Ciencias Básicas, Universidad del Atlántico, Puerto Colombia 081001, Colombia

[5] Grupo de Investigaciones en Química Y Biología, Departamento de Química Y Biología, Facultad de Ciencias Básicas, Universidad del Norte, Carrera 51B, Km 5, Vía Puerto Colombia, Barranquilla 081007, Colombia

* Correspondence: jhernandezf@unicartagena.edu.co (J.H.-F.); ebrazon@uninorte.edu.co (E.M.);

Tel.: +57-301-562-4990 (J.H.-F.)

All simulations were conducted using Density Functional Theory (DFT) and Gaussian 16 software. Geometry optimizations were performed using the B3LYP functional, which stands for Becke's three-parameter hybrid exchange-correlation functional (D. M. Becke) and Lee, Yang, and Parr (B3LYP), in reference to their respective contributors (Lee, Yang, and Parr) in the development of Density Functional Theory (DFT). This functional is widely utilized in computational chemistry and DFT calculations due to its ability to provide accurate results across a broad range of chemical systems.

The electronic configurations of atoms are described using a triple-ξ basis set with a polarization function (Gaussian 6-311G basis set). After optimizing molecular structures, detailed energy calculations were performed using the B3LYP/6-311G method on the resulting geometries. The energy values obtained with this method are presented in this report because, as previously demonstrated, this approach yields results closer to experimental data. Moreover, the B3LYP method is widely recognized and employed in simulating catalytic systems involving transition metals.

List of Cartesian coordinates

Ziegler–Natta catalyst

Mg	2.68393000	2.81002700	0.00044400
Mg	4.14706300	-0.74690400	0.02993200
Mg	5.58907800	-4.15634900	0.30013100
Mg	6.49066600	2.27953800	0.07571300
Mg	7.97624200	-1.30379900	0.20607100
Mg	-1.08695500	3.09817400	-0.12319800
Mg	0.40675100	-0.42804800	-0.01870400
Mg	1.90262200	-4.00134000	0.09581200
Mg	-3.36174400	0.04345300	-0.11804000
Mg	-1.87914600	-3.57154500	-0.03412600
Mg	-5.66354900	-3.07014400	-0.03015900
Mg	-7.05937700	0.48185200	-0.35091800
Mg	-9.36716100	-2.34535900	0.29277000
Mg	10.19359400	1.66175700	-0.26708100
Cl	4.39995200	1.36855100	-1.35036000
Cl	4.73681200	3.85172100	0.80417600
Cl	0.86384200	4.36605100	0.58559600
Cl	0.68407500	1.73023200	-1.43622900

Cl	2.36210100	0.59646700	1.29938400
Cl	5.86816900	-2.18790500	-1.22545200
Cl	6.14593700	0.06631900	1.39961200
Cl	2.18406900	-1.82718300	-1.24466300
Cl	3.83072200	-2.86789800	1.55437300
Cl	7.78734100	-3.66682300	1.09193000
Cl	3.79179600	-5.55872100	-0.45100200
Cl	8.00255200	0.72579100	-1.41822400
Cl	8.59199200	3.18590300	0.84169700
Cl	10.11014200	-0.50088200	0.96020100
Cl	-3.03294300	2.16245200	-1.62831800
Cl	-1.37817200	0.97215000	1.21238400
Cl	-1.56745500	-1.37105200	-1.35311300
Cl	0.12510200	-2.57459500	1.39903800
Cl	-0.09496400	-5.13496400	-0.70468500
Cl	-5.08591000	1.54039800	1.01905600
Cl	-5.28241800	-0.93768000	-1.47882100
Cl	-3.58744000	-2.06719200	1.31145500
Cl	-3.94227800	-4.69093300	-0.68944300
Cl	-7.14102200	-1.42397800	1.39189400
Cl	-7.79712800	-3.92495200	-0.78243400
Cl	-9.25705900	-0.20249100	-0.99479200
Cl	11.99496800	2.33961200	-1.45277600
Cl	-11.18072900	-2.94813000	1.49773300
Cl	-2.98249700	4.38974500	0.80299400
Cl	-6.91249400	2.72792500	-1.39939600
Cl	-4.80181600	5.43274400	-1.98005000
Ti	-4.79879500	3.65637300	-0.66922200

Furane

C	-0.54841400	0.99723800	-0.00010400
C	0.81207300	1.00595200	0.00008200
C	1.22508400	2.38876100	-0.00003800
C	0.09219000	3.14212400	-0.00013100
O	-1.02608700	2.30804600	0.00005900
H	-1.29769200	0.23078600	-0.00015400
H	1.45738200	0.14570200	0.00017300
H	2.23646500	2.75422600	-0.00004500
H	-0.11395600	4.19396700	-0.00020200

$MgCl_2$-$TiCl_4$-Furane

Mg	2.68393000	2.81002800	0.00043900
Mg	4.14706300	-0.74690400	0.02993200
Mg	5.58907800	-4.15634900	0.30013100
Mg	6.49066600	2.27953800	0.07571300
Mg	7.97624300	-1.30379900	0.20607100
Mg	-1.08696700	3.09846500	-0.12312900
Mg	0.40674400	-0.42803700	-0.01873000
Mg	1.90262200	-4.00134000	0.09581200
Mg	-3.36187400	0.04341000	-0.11820800
Mg	-1.87912900	-3.57153800	-0.03411100
Mg	-5.66361200	-3.07017600	-0.03019700
Mg	-7.05772900	0.48080800	-0.35052700
Mg	-9.36720600	-2.34531300	0.29281400
Mg	10.19359500	1.66175700	-0.26708100
Cl	4.39995200	1.36855100	-1.35036000
Cl	4.73681200	3.85172100	0.80417600
Cl	0.86399300	4.36585000	0.58554200
Cl	0.68407200	1.73023100	-1.43623000
Cl	2.36210100	0.59646700	1.29938400

Cl	5.86816900	-2.18790500	-1.22545200
Cl	6.14593700	0.06631900	1.39961200
Cl	2.18406900	-1.82718300	-1.24466300
Cl	3.83072200	-2.86789800	1.55437300
Cl	7.78734200	-3.66682300	1.09193000
Cl	3.79179600	-5.55872100	-0.45100200
Cl	8.00255300	0.72579100	-1.41822400
Cl	8.59199300	3.18590300	0.84169700
Cl	10.11014300	-0.50088200	0.96020100
Cl	-3.03343800	2.16261000	-1.62811000
Cl	-1.37815300	0.97211900	1.21231800
Cl	-1.56746000	-1.37107000	-1.35309900
Cl	0.12510200	-2.57459500	1.39903800
Cl	-0.09496400	-5.13496400	-0.70468500
Cl	-5.08630600	1.54093300	1.01883700
Cl	-5.28243500	-0.93765600	-1.47891600
Cl	-3.58742200	-2.06717900	1.31147800
Cl	-3.94227800	-4.69093300	-0.68944300
Cl	-7.14164100	-1.42397100	1.39187200
Cl	-7.79712900	-3.92495200	-0.78243400
Cl	-9.25712500	-0.20221600	-0.99486200
Cl	11.99496900	2.33961200	-1.45277600
Cl	-11.18073000	-2.94813000	1.49773300
Cl	-3.02381400	4.27295300	0.99058600
Cl	-6.86343900	2.69301000	-1.43906500
Cl	-4.85931600	5.51408800	-1.74723500
Ti	-4.77074300	3.63439000	-0.58162900
C	-0.96374900	4.31263500	-3.11878100
C	-0.88079900	5.50157800	-3.75185500
C	-1.01544500	6.54729600	-2.76086800
C	-1.17405300	5.96207900	-1.55588200
O	-1.15201400	4.54800000	-1.72623800
H	-0.92148500	3.28478000	-3.41621300
H	-0.74212800	5.64132400	-4.80873200
H	-0.99764600	7.60583000	-2.94691200
H	-1.30330100	6.30981700	-0.55090400

$TiCl_4$

Ti	0.17891	0.02825	0.
Cl	0.94892	1.11718	1.88611
Cl	0.94888	-2.14965	0.
Cl	0.94892	1.11718	-1.88611
Cl	-2.13109	0.02828	0.

Orbital energies and kinetic energies of $TiCl_4$ (alpha):

		1	2
1	O	-179.037358	231.847693
2	O	-101.574043	136.906749
3	O	-101.574039	136.906781
4	O	-101.573966	136.906695
5	O	-101.573965	136.906845
6	O	-20.075876	40.567531
7	O	-16.789417	39.439718
8	O	-16.789415	39.439719
9	O	-16.789414	39.439722
10	O	-9.492630	21.544415
11	O	-9.492616	21.544603
12	O	-9.492540	21.544497
13	O	-9.492530	21.544625

14	O	-7.251672	20.537600
15	O	-7.251664	20.538347
16	O	-7.251587	20.537448
17	O	-7.251581	20.538895
18	O	-7.248929	20.548014
19	O	-7.248926	20.548563
20	O	-7.248915	20.549487
21	O	-7.248912	20.550403
22	O	-7.248841	20.548004
23	O	-7.248839	20.548561
24	O	-7.248823	20.551772
25	O	-7.248820	20.551971
26	O	-2.512921	8.187089
27	O	-1.621042	6.832505
28	O	-1.621029	6.832581
29	O	-1.621018	6.832671
30	O	-0.847128	3.146445
31	O	-0.836084	3.276643
32	O	-0.836032	3.276473
33	O	-0.835981	3.276324
34	O	-0.420661	2.423180
35	O	-0.409026	2.671677
36	O	-0.408976	2.671277
37	O	-0.408926	2.670957
38	O	-0.391716	2.220005
39	O	-0.391711	2.220018
40	O	-0.378262	2.195072
41	O	-0.378237	2.195003
42	O	-0.378207	2.194935
43	O	-0.346306	2.433491
44	O	-0.346286	2.433391
45	O	-0.346263	2.433352
46	V	-0.160543	3.950402
47	V	-0.160538	3.950425
48	V	-0.136306	3.896470
49	V	-0.136296	3.896324
50	V	-0.136282	3.896227
51	V	-0.009526	0.667792
52	V	0.033321	0.508849
53	V	0.033326	0.508813
54	V	0.033341	0.508818
55	V	0.150734	1.857188
56	V	0.184602	2.050740
57	V	0.184613	2.050646
58	V	0.184668	2.050857
59	V	0.312847	1.469119
60	V	0.393078	2.762256
61	V	0.393096	2.762238
62	V	0.397747	2.652776
63	V	0.397755	2.652655
64	V	0.397768	2.652545
65	V	0.500572	2.787019
66	V	0.500577	2.786893
67	V	0.500584	2.786952
68	V	0.507431	2.812474
69	V	0.522555	2.531453
70	V	0.522592	2.531518
71	V	0.522616	2.531729
72	V	0.562611	2.486443
73	V	0.562635	2.486349
74	V	0.562653	2.486125
75	V	0.609710	3.519196
76	V	0.609722	3.519157

```
 77         V                 0.768623          3.599164
 78         V                 0.768707          3.598847
 79         V                 0.768827          3.598452
 80         V                 0.784164          2.801796
 81         V                 0.805169          2.626330
 82         V                 0.805202          2.626325
 83         V                 0.805235          2.626323
 84         V                 0.833322          2.627013
 85         V                 0.833327          2.626995
 86         V                 0.833621          2.628099
 87         V                 0.833647          2.628071
 88         V                 0.833666          2.628087
 89         V                 0.840435          2.747796
 90         V                 0.840462          2.747937
 91         V                 0.840512          2.748288
 92         V                 1.002297          3.061026
 93         V                 1.002355          3.060865
 94         V                 1.002404          3.061146
 95         V                 1.083828          2.909579
 96         V                 1.129886          3.624229
 97         V                 1.129899          3.624251
 98         V                 1.344327          3.716542
 99         V                 1.344342          3.716447
100         V                 1.344352          3.716360
101         V                 1.976852          3.654937
102         V                 1.976900          3.654988
103         V                 1.976915          3.655024
104         V                 2.026256          3.869361
105         V                 2.026308          3.869694
106         V                 2.026390          3.870032
107         V                 2.161727          3.962810
108         V                 4.224045         14.870735
109         V                 4.224066         14.870753
110         V                 4.224076         14.870724
111         V                 4.244815         14.537805
112         V                 5.186209          9.379947
Total kinetic energy from orbitals= 2.683023492946D+03
```

Furan orbital symmetries:

```
      Occupied  (A) (A) (A) (A) (A) (A) (A) (A) (A) (A) (A) (A)
                (A) (A) (A) (A) (A) (A)
      Virtual   (A) (A) (A) (A) (A) (A) (A) (A) (A) (A) (A) (A)
                (A) (A) (A) (A) (A) (A) (A) (A) (A) (A) (A) (A)
                (A) (A) (A) (A) (A) (A) (A) (A) (A) (A) (A) (A)
                (A) (A) (A) (A) (A) (A) (A) (A) (A) (A) (A) (A)
                (A) (A) (A) (A) (A) (A) (A) (A) (A) (A) (A) (A)
                (A) (A) (A) (A) (A)
The electronic state is 1-A.
Alpha  occ. eigenvalues --  -19.21133 -10.23164 -10.23162 -10.18441 -10.18400
Alpha  occ. eigenvalues --   -1.10325  -0.79265  -0.73937  -0.59314  -0.56552
Alpha  occ. eigenvalues --   -0.53650  -0.44173  -0.44170  -0.40765  -0.39336
Alpha  occ. eigenvalues --   -0.36673  -0.27346  -0.22446
Alpha virt. eigenvalues --    0.01968   0.07854   0.11754   0.14136   0.15499
Alpha virt. eigenvalues --    0.16640   0.19596   0.20562   0.31421   0.37861
Alpha virt. eigenvalues --    0.43335   0.51524   0.53358   0.58138   0.58180
Alpha virt. eigenvalues --    0.58518   0.59287   0.61487   0.62906   0.67559
Alpha virt. eigenvalues --    0.67949   0.80098   0.82841   0.84199   0.90760
Alpha virt. eigenvalues --    0.90838   0.98223   0.98778   1.01655   1.03533
Alpha virt. eigenvalues --    1.13400   1.13870   1.34885   1.37562   1.38539
Alpha virt. eigenvalues --    1.39393   1.53567   1.55389   1.56626   1.57378
Alpha virt. eigenvalues --    1.68078   1.84147   1.93158   2.01352   2.05462
Alpha virt. eigenvalues --    2.08610   2.19764   2.21966   2.25993   2.26051
```

```
Alpha virt. eigenvalues --    2.32028   2.39875   2.57389   2.62504   2.62911
Alpha virt. eigenvalues --    2.70320   2.78274   2.93198   2.94843   3.10078
Alpha virt. eigenvalues --    3.93769   4.14799   4.14936
```
